# Anatomical and Functional Outcomes of Repeat Vitrectomy Combined with Encircling Scleral Buckling for First Recurrent Rhegmatogenous Retinal Detachment After Primary Vitrectomy: A Retrospective Cohort Study

**DOI:** 10.3390/jcm15176801

**Published:** 2026-09-02

**Authors:** Georgios D. Panos, Alex Stubbing-Moore, Dharmalingam Kumudhan, Gavin Orr, Anwar Zaman, Craig Wilde

**Affiliations:** 1 Department of Ophthalmology, Queen’s Medical Centre, Nottingham University Hospitals, Derby Road, Lenton, Nottingham NG7 2UH, UK; 2 Division of Ophthalmology and Visual Sciences, School of Medicine, Faculty of Medicine & Health Sciences, University of Nottingham, Nottingham NG7 2UH, UK

**Keywords:** rhegmatogenous retinal detachment, recurrent retinal detachment, pars plana vitrectomy, encircling scleral buckle, proliferative vitreoretinopathy, silicone oil, retinal reattachment, vitreoretinal surgery

## Abstract

**Purpose**: The purpose of this study is to evaluate anatomical, visual, and safety outcomes after repeat pars plana vitrectomy combined with 360° encircling scleral buckling for first recurrent rhegmatogenous retinal detachment following primary vitrectomy. **Methods**: This retrospective single-centre cohort included 47 eyes of 47 patients treated between October 2016 and October 2023. The primary outcome was freedom from any subsequent retinal re-detachment during available follow-up. Secondary outcomes included final best-corrected visual acuity, timing, and management of further re-detachment, silicone oil management, and postoperative complications. Exploratory analyses described break location and final visual outcomes according to selected clinical characteristics. **Results**: The median age was 57.6 years, and the median follow-up was 13 months. Freedom from any subsequent retinal re-detachment was achieved in 41/47 eyes (87.2%; 95% CI 74.8–94.0%). The median final visual acuity was 0.48 logMAR. Six eyes developed a subsequent re-detachment; four required further vitrectomy with retinectomy. Among 18 break-related first recurrences, 12 involved an inferior, inferonasal, or inferotemporal break, and no subsequent re-detachment was documented in these 12 eyes during available follow-up. Clinically relevant complications occurred in 11 eyes, including treated intraocular pressure elevation in seven and buckle exposure in four. **Conclusions**: Repeat vitrectomy with encircling scleral buckling was followed by freedom from subsequent retinal re-detachment in 87.2% of eyes. The descriptive break location findings identify inferior break-related recurrence as a candidate phenotype for future comparative evaluation, but the absence of a repeat PPV-only comparator precludes attribution of incremental benefit to the buckle.

## 1. Introduction

Rhegmatogenous retinal detachment (RRD) is a sight-threatening condition that requires timely surgical repair. Pars plana vitrectomy (PPV) has become a principal surgical approach for primary RRD, particularly in pseudophakic eyes and detachments with complex or posterior pathology [1]. Nevertheless, primary repair may fail, and recurrent RRD remains a challenging clinical problem associated with further surgical intervention, cumulative morbidity, and an increased risk of poor visual outcome [1,2]. Re-detachment after PPV may result from missed, persistent, or newly formed retinal breaks, inadequate relief of vitreoretinal traction, or the development of proliferative vitreoretinopathy (PVR), which remains one of the most important causes of surgical failure [1,3].

The management of recurrent RRD after primary PPV is individualised according to the anatomical cause and extent of the recurrence, macular status, presence, and severity of PVR, location of the retinal breaks, lens status, and previous tamponade. Repeat PPV is generally central to the management of these eyes and may be combined with further vitreous-base shaving, membrane peeling, retinotomy or retinectomy, additional retinopexy, and gas or silicone oil tamponade [1]. An encircling scleral buckle may also be added during reoperation. By supporting the peripheral retina and vitreous base, reducing circumferential and anteroposterior traction, and providing support for occult, anterior or inferior retinal breaks, an encircling buckle may offer a useful adjunct in selected post-vitrectomy eyes. However, buckle placement is associated with additional operative complexity and potential adverse effects, including induced refractive change, ocular discomfort, buckle exposure or infection, and the possible need for subsequent buckle removal [1,4].

Evidence concerning the role of scleral buckling in recurrent RRD after previous vitrectomy remains limited and heterogeneous. Small retrospective series have reported favourable anatomical outcomes following scleral buckling, with or without gas tamponade, in selected post-vitrectomy eyes [4]. Conversely, repeat vitrectomy without an additional buckle has also achieved satisfactory retinal reattachment in selected cases [5]. In eyes with recurrent detachment secondary to PVR, one retrospective comparative study reported greater anatomical success with repeat PPV combined with scleral buckling than with repeat PPV alone, although the non-randomised design and potential differences in case selection limit causal interpretation [6]. More recently, a cohort of 127 eyes with recurrent RRD found a second-surgery success rate of 75% and no difference in anatomical or functional outcomes according to whether the scleral buckle was placed during the primary or secondary operation [2]. Comparisons among published studies are further complicated by differences in baseline complexity, PVR severity, tamponade selection, follow-up duration, and the definition of anatomical success, particularly whether retinal attachment under retained silicone oil is considered a successful outcome [2,4,5,6].

There, therefore, remains a need for detailed real-world outcome data from clinically well-defined cohorts undergoing a consistent reoperation strategy. The present study evaluated the anatomical and functional outcomes of repeat PPV combined with encircling scleral buckling for the first recurrence of RRD following primary PPV. Secondary objectives were to describe further retinal procedures, postoperative complications, and the clinical phenotype of the first recurrence, including documented retinal break location. As all included eyes received an encircling buckle during reoperation, the study was designed to characterise outcomes following this combined approach rather than to estimate the independent treatment effect of the buckle.

## 2. Materials and Methods

### 2.1. Study Design and Setting

This retrospective, single-centre observational cohort study included patients undergoing repeat pars plana vitrectomy (PPV) combined with an encircling scleral buckle for the first recurrence of rhegmatogenous retinal detachment (RRD) following primary PPV at the Department of Ophthalmology at Queen’s Medical Centre, Nottingham, UK. The repeat procedures were performed between October 2016 and October 2023. The study was reported in accordance with the Strengthening the Reporting of Observational Studies in Epidemiology (STROBE) recommendations for cohort studies [7].

The study was approved by the Clinical Effectiveness Department under reference 23-744C. The study was conducted in accordance with the principles of the Declaration of Helsinki.

### 2.2. Study Population

Eyes were eligible if they had undergone primary PPV for RRD, subsequently developed a first recurrent retinal detachment requiring further surgery, and were treated at the second operation with repeat PPV combined with an encircling scleral buckle. The primary PPV could have been performed at the study centre or elsewhere, provided that sufficient clinical information was available for data extraction.

Eyes were excluded if the encircling buckle was placed only during a third or subsequent retinal detachment operation, if the clinical episode could not be reliably reconstructed, or if the record represented a duplicate entry. No minimum postoperative follow-up duration was imposed. Each patient contributed only one eye to the analysis; therefore, no adjustment for inter-eye correlation was required.

### 2.3. Surgical Management

All included eyes underwent repeat 23-gauge PPV combined with placement of a 360° encircling 276 silicone tyre using the technique previously described by Wilde et al. [8]. The procedures were performed by two consultant vitreoretinal surgeons at the study centre. Residual vitreous and vitreoretinal traction were addressed according to the intraoperative findings, with internal drainage of subretinal fluid. Retinopexy was applied directly to the identified retinal breaks using endolaser photocoagulation, trans-scleral cryotherapy, or a combination of both, according to the intraoperative findings. Routine prophylactic 360° laser retinopexy was not performed. Additional membrane peeling, retinotomy, or lens surgery was performed when clinically indicated. Internal tamponade consisted of 12% C3F8 or 1000-centistoke silicone oil. The choice of tamponade and supplementary procedures was based on retinal configuration, break location, PVR, and the operating surgeon’s clinical judgement; the timing of subsequent silicone oil removal was individualised.

### 2.4. Data Collection

Data were retrospectively extracted from Medisoft and Medisight electronic medical records, operative records, and vitreoretinal theatre databases using a standardised data-collection form. The variables recorded included age at the second operation, operated eye, dates of the primary and repeat operations, lens status, duration and macular status of the primary detachment, the documented cause of the primary and recurrent detachments, PVR, preoperative and postoperative intraocular pressure (IOP), tamponade used during each operation, removal of silicone oil, cataract or lens surgery, postoperative complications, further retinal procedures, and duration of follow-up.

The interval between the primary and repeat operations was calculated in days, and age was calculated at the date of the repeat operation. The cause of the first recurrent detachment was based on the treating vitreoretinal surgeon’s documentation. For analysis, PVR was classified only when it was clearly documented as the anatomical cause of the first recurrence; PVR was not inferred from other clinical features. PVR grade and anatomical extent were not consistently documented and were therefore not used for classification. Failure to follow postoperative positioning instructions was recorded as a potential contributing factor rather than as a structural cause. For the post hoc break location description, break-related recurrences were categorised from the source record as inferior/inferonasal/inferotemporal, nasal, superior/superonasal/superotemporal, or location not specified. Variables not recorded in the clinical documentation were treated as missing and were not recoded as absence of the characteristic.

### 2.5. Visual Acuity Assessment

Best-corrected visual acuity (BCVA) was recorded in logMAR. Low-vision categories were assigned the following conventional logMAR values: counting fingers, 1.90; hand movements, 2.30; and perception of light, 2.70 [9]. The postoperative BCVA after the second operation was defined as the final recorded BCVA at the last available follow-up visit.

Because the timing of the BCVA recorded after the primary operation was not uniform and could not be confirmed in every eye as the measurement immediately preceding the second operation, change from this measurement to final postoperative BCVA was considered exploratory rather than a prespecified principal functional outcome.

### 2.6. Outcomes

The primary outcome was freedom from any documented subsequent retinal re-detachment following the second operation until the last recorded follow-up. Subsequent retinal re-detachment was defined as any new area of detached retina after the combined procedure, irrespective of its extent or subsequent management. One event represented a limited re-detachment that was managed with additional laser retinopexy without further retinal reattachment surgery.

A sensitivity definition of anatomical success was also examined in which this limited, laser-treated re-detachment was not classified as failure of the repeat surgical procedure because it did not require further PPV or other major retinal reattachment surgery.

Secondary outcomes included final BCVA, time to subsequent retinal re-detachment, re-detachment while silicone oil remained in situ, re-detachment following removal of silicone oil, further PPV, retinectomy, silicone oil removal, and documented postoperative complications. Complications of particular interest included postoperative IOP elevation requiring treatment, buckle exposure, buckle removal or other buckle-related intervention, and migration of silicone oil into the anterior chamber.

### 2.7. Statistical Analysis

No formal sample size calculation was performed because all eligible eyes available during the study period were included. Continuous variables were summarised using the median, interquartile range (IQR), and range, and categorical variables were presented as frequencies and percentages. Ninety-five per cent confidence intervals (CIs) for proportions were calculated using the Wilson score method.

The primary anatomical outcome was calculated for the complete cohort. The post hoc break location analysis was descriptive; no formal hypothesis testing was applied to these small phenotype subgroups.

As a sensitivity analysis addressing limited postoperative observation, the primary anatomical outcome was recalculated after excluding eyes with less than 6 months of available follow-up. This restricted cohort analysis was not interpreted as a fixed 6-month or actuarial re-detachment-free estimate.

Exploratory comparisons of final BCVA between independent groups were performed using the Mann–Whitney U test. These analyses were unadjusted, and no correction for multiple comparisons was applied.

Time to subsequent retinal re-detachment was summarised descriptively. A formal Kaplan–Meier analysis was not undertaken because the timing of one of the six events was unavailable.

Missing data were analysed using available case analysis, with denominators reported for each analysis. No missing values were imputed. All statistical tests were two-sided, and a *p* value below 0.05 was considered statistically significant. Exploratory visual findings were interpreted cautiously in view of the small subgroup sizes and lack of multiplicity adjustment.

Statistical analyses were performed using R version 4.6.1 (R Foundation for Statistical Computing, Vienna, Austria). Data were imported using the readxl package version 1.5.0.

## 3. Results

### 3.1. Study Cohort

The study included 47 eyes from 47 patients (Table 1). Twenty-six eyes (55.3%) were right eyes, and 21 (44.7%) were left eyes. The median age at the second operation was 57.6 years (IQR 52.6–67.2; range 36.7–81.4 years). The median interval between the primary and repeat operations was 42 days (IQR 25.5–63.5; range 10–940 days).

The median duration of follow-up after the second operation was 13.0 months (IQR 6.5–24.0; range 1–77 months). Twenty-three eyes (48.9%) received C3F8 tamponade during the second operation, and 24 (51.1%) received silicone oil. The cause or contributing factor for the first recurrence was documented in 35 eyes: 18/35 (51.4%) were break-related, 14/35 (40.0%) were attributed to PVR, 2/35 (5.7%) had another documented cause, and 1/35 (2.9%) had non-adherence to postoperative positioning recorded as a contributing factor; cause was missing in 12 eyes. Macular status at recurrence was documented in 35 eyes, of which 19/35 (54.3%) were macula-on and 16/35 (45.7%) were macula-off.

### 3.2. Anatomical Outcomes

During follow-up, no subsequent retinal re-detachment was documented in 41 of 47 eyes, corresponding to a primary anatomical success rate of 87.2% (95% CI 74.8–94.0%) (Table 2). Six eyes developed a subsequent re-detachment after the second operation.

One of the six events consisted of a limited re-detachment successfully managed with additional laser retinopexy without further PPV. When this event was not classified as surgical failure, anatomical success was achieved in 42 of 47 eyes (89.4%; 95% CI 77.4–95.4%) (Table 2).

In a sensitivity analysis restricted to the 38 eyes with at least 6 months of available follow-up, 32 remained free from any subsequent retinal re-detachment (84.2%; 95% CI 69.6–92.6%), consistent with the primary full cohort estimate.

The timing of subsequent retinal re-detachment was available for five of the six affected eyes. Events occurred at 2, 2, 6, 8, and 33 months after the second operation, giving a median known time to re-detachment of 6 months (IQR 2–8 months). Four of the five events with known timing occurred within eight months, whereas one occurred at 33 months. The timing of the remaining event was not documented.

Four subsequent re-detachments were managed with further PPV and retinectomy. The limited re-detachment was treated with laser retinopexy alone, while the subsequent management of one eye was incompletely documented. Three re-detachments occurred while silicone oil remained in situ, and one occurred following silicone oil removal. Clinical details of the six subsequent retinal re-detachments are provided in Supplementary Table S1.

### 3.3. Break Location Phenotype

Among the 18 eyes with a break-related first recurrence, 12 (66.7%) had an inferior, inferonasal, or inferotemporal break, two (11.1%) had a nasal break, three (16.7%) had a superior, superonasal, or superotemporal break, and one (5.6%) had inadequately treated tears without a documented location (Table 1). No subsequent retinal re-detachment was documented among the 12 eyes with inferior/inferonasal/inferotemporal breaks during a median follow-up of 11.5 months (IQR 7.8–18.3; range 1–77 months). Across all 18 break-related recurrences, 17 eyes remained free from any subsequent re-detachment; the single event was the limited re-detachment managed with laser retinopexy alone. Among the 14 eyes in which PVR was documented as the cause of the first recurrence, four developed a subsequent re-detachment.

### 3.4. Silicone Oil Outcomes

Among the 24 eyes receiving silicone oil tamponade during the second operation, straightforward silicone oil removal was documented in 15 eyes (Table 3). The median time to oil removal was 4 months. Silicone oil remained in situ at the final recorded follow-up in eight eyes. One additional eye underwent silicone oil exchange for heavy silicone oil following a subsequent retinal re-detachment, followed by subsequent oil removal.

A subsequent retinal re-detachment occurred while silicone oil remained in situ in three of the 24 oil-treated eyes. One re-detachment occurred after documented silicone oil removal. These findings were descriptive because the timing and duration of follow-up after oil removal were not standardised.

### 3.5. Visual Outcomes

The median final recorded BCVA after the second operation was 0.48 logMAR (IQR 0.24–0.81; range −0.08 to 2.70 logMAR) (Table 2).

In exploratory analyses, eyes receiving silicone oil had worse final BCVA than those receiving C3F8, with median values of 0.64 logMAR (IQR 0.38–0.86) and 0.30 logMAR (IQR 0.14–0.70), respectively (Mann–Whitney *p* = 0.018) (Table 4). This comparison was unadjusted and susceptible to confounding by indication, as silicone oil was preferentially used in more complex detachments.

Eyes with macula-off first recurrence had a median final BCVA of 0.72 logMAR (IQR 0.47–0.95), compared with 0.30 logMAR (IQR 0.18–0.63) among eyes with macula-on recurrence (*p* = 0.011) (Table 4). Twelve eyes with undocumented macular status were excluded from this comparison.

Median final BCVA was 0.62 logMAR (IQR 0.34–1.02) in eyes with PVR and 0.52 logMAR (IQR 0.24–0.71) in eyes with another documented anatomical cause of the first recurrence (*p* = 0.214) (Table 4). Eyes with missing cause of detachment data and the eye with non-adherence to positioning as the only documented contributing factor were excluded from this comparison.

### 3.6. Postoperative Complications

At least one clinically relevant postoperative complication was documented in 11 eyes (23.4%). Postoperative IOP elevation requiring treatment occurred in seven eyes (14.9%). Buckle exposure was documented in four eyes (8.5%), and five eyes (10.6%) underwent buckle removal or another buckle-related intervention. In one eye (2.1%), silicone oil entered the anterior chamber and was removed three days after surgery.

## 4. Discussion

In this retrospective cohort of eyes undergoing surgery for a first recurrent rhegmatogenous retinal detachment after primary PPV, repeat PPV combined with a 360° encircling scleral buckle was followed by freedom from any subsequent retinal re-detachment in 87.2% of eyes. When the limited re-detachment was managed successfully with additional laser retinopexy alone and was not classified as failure of the repeat operation, the anatomical success proportion was 89.4%. Median final BCVA was 0.48 logMAR, and six eyes developed a subsequent re-detachment, of which four required repeat PPV with retinectomy. A post hoc descriptive analysis also identified a predominance of inferior break-related pathology among break-related first recurrences. These findings characterise outcomes after a consistent combined reoperation strategy but do not isolate the independent contribution of the buckle.

Contemporary evidence specifically addressing the management of recurrent RRD after primary PPV remains limited. In a retrospective cohort of 127 eyes undergoing surgery for recurrent RRD, Hébert et al. reported a second surgery success rate of 75% in both eyes treated with PPV followed by combined PPV and scleral buckling and those treated with combined surgery initially followed by PPV at recurrence [2]. Among the 51 eyes treated with primary PPV followed by combined PPV and scleral buckling, 38 achieved second surgery success (75%) [2]. Their definition of success was the absence of further operative intervention and therefore differed from our conservative primary definition, which classified the limited laser-treated re-detachment as an event. When this event was excluded from our failure definition, the anatomical success proportion increased to 89.4%. Differences in patient selection, severity of PVR, operative technique, duration of follow-up, and outcome definitions preclude direct comparison or any conclusion that one cohort achieved superior results.

Our findings are also broadly consistent with a smaller study specifically examining encircling buckling after previous vitreoretinal surgery. Shishkin et al. evaluated 21 eyes with recurrent RRD following primary vitrectomy and reported anatomical success in 95% after circular episcleral buckling, either alone or in combination with revision of the vitreous cavity [10]. That cohort was selected for recurrence associated with PVR progression and differed from ours in surgical management, case selection, and outcome assessment. Nevertheless, the two studies support the feasibility of adding an encircling element when recurrence follows an initial vitrectomy.

There are several potential mechanisms by which an encircling buckle may complement repeat PPV in recurrent RRD. Encirclement provides circumferential support to the vitreous base, reduces residual peripheral traction, and may support small, occult, or newly formed retinal breaks. This mechanical effect may be particularly relevant to inferior peripheral pathology, where residual vitreous base traction and tamponade geometry can complicate repair. Repeat vitrectomy simultaneously permits identification and treatment of missed or new breaks, removal of residual vitreous traction, membrane dissection and, when required, retinotomy or retinectomy.

Evidence from primary RRD surgery suggests that the effect of adding a scleral buckle depends on case selection. In a propensity-matched cohort of primary uncomplicated RRD, the addition of a buckle to PPV did not significantly improve single-surgery anatomical success [11]. Conversely, combined PPV and scleral buckling was associated with higher single-surgery success in eyes considered at high risk of postoperative PVR [12] and in a multicentre cohort with inferior retinal breaks, particularly among phakic eyes [13]. Echegaray et al. similarly reported greater single-operation anatomical success with combined PPV and buckling than with PPV alone in primary RRD, with the difference concentrated in phakic eyes [14]. Although these studies concern primary rather than recurrent RRD, they support selective rather than universal use of an additional buckle.

The break location distribution in the present cohort provides a hypothesis for future case selection. Two-thirds of break-related first recurrences involved an inferior, inferonasal, or inferotemporal break, and no subsequent retinal re-detachment was documented among these 12 eyes during available follow-up. This pattern is compatible with the mechanical rationale for encirclement, but the subgroup was small, follow-up was variable, and all eyes received a buckle; it, therefore, cannot establish an incremental treatment effect. In a retrospective series of inferior recurrent RRD, Churashov et al. reported a lower recurrence rate after combined PPV and scleral buckling for first inferior recurrence than after several alternative reoperations [15]. A recent systematic review of inferior break RRD found the comparative evidence to be mixed overall, although a single-surgery anatomical advantage with supplemental buckling was observed in phakic eyes in subgroup analysis [16]. Taken together, these data support further comparative evaluation of discrete inferior break-related recurrence as a candidate phenotype rather than a definitive indication for combined surgery.

PVR was documented as the cause of the first recurrence in 14 eyes, of which four developed a subsequent re-detachment. This descriptive pattern is consistent with PVR as a marker of greater retinal complexity, but PVR grade and extent were not consistently available. Ambiya et al. reported that grade C or greater PVR and the need for multiple repeat procedures were associated with anatomical failure after surgery for recurrent retinal detachment [17]. The present data, therefore, support careful prognostic interpretation of PVR but cannot determine whether PVR modifies the incremental benefit of adding a buckle.

Visual recovery after recurrent RRD surgery depends on factors beyond final retinal attachment, including macular involvement, duration of detachment, previous macular injury, PVR, cumulative surgical trauma, tamponade-related effects, and postoperative macular pathology. Eyes presenting with macula-off recurrent detachment in our cohort had worse final BCVA than those with macula-on recurrence. This finding is clinically plausible, but macular status was missing in 12 eyes, BCVA immediately before the second operation was not uniformly available, and the analysis was unadjusted. The observed association should, therefore, be regarded as exploratory rather than as a quantified independent effect.

Eyes treated with silicone oil had worse final BCVA than those treated with C3F8. This finding is particularly susceptible to confounding by indication because silicone oil was preferentially selected for eyes with greater retinal complexity, including PVR, extensive pathology, retinectomy, or concern regarding postoperative positioning. The worse visual outcome should therefore not be attributed to silicone oil itself.

In the recurrent RRD cohort reported by Hébert et al., silicone oil was used during the second operation in 35% of eyes treated with PPV followed by combined PPV and scleral buckling and in 47% of those treated with combined surgery followed by PPV [2]. Retinal re-detachment can also occur following silicone oil removal, particularly in eyes with previous failed surgery or complex PVR. A retrospective cohort reported retinal re-detachment in 14.9% of eyes after silicone oil removal for RRD and identified previous unsuccessful retinal surgery as an important risk factor [18]. These observations reinforce the interpretation that silicone oil use and oil-related outcomes primarily reflect underlying retinal complexity rather than an independently comparable treatment exposure.

Most subsequent re-detachments with known timing occurred relatively early, with four of five events occurring within eight months. However, one eye developed re-detachment at 33 months. Late recurrent RRD is uncommon but well recognised. Anguita et al. identified 39 late recurrences among more than 16,000 retinal detachment operations and reported that these cases were frequently associated with small retinal breaks that could be difficult to identify [19]. Foster et al. similarly described recurrent retinal detachment occurring more than one year after apparently successful repair, with some recurrences arising several years after the initial operation [20]. The late event in our cohort supports continued patient education regarding recurrent symptoms even after prolonged anatomical stability. The absence of a documented event time in one eye and variable follow-up precluded reliable time-to-event estimation.

Clinically relevant postoperative complications were documented in 11 eyes, including IOP elevation requiring treatment in seven, buckle exposure in four, and buckle intervention or removal in five. Exposure, infection, discomfort, and ocular motility disturbance are recognised indications for scleral buckle removal. In a series examining buckle removal, most symptomatic patients experienced improvement following removal, although recurrent retinal detachment and other complications remained possible [21]. Kim et al. reported retinal re-detachment in four of 40 eyes following scleral buckle removal, with encircling buckle removal associated with a greater recurrence risk than removal of a segmental element [22]. The observed complication frequencies should be interpreted in the context of variable follow-up and retrospective ascertainment.

This study has several strengths. It examined a narrowly defined and clinically relevant population: first recurrent RRD after primary PPV, with all eyes undergoing repeat PPV combined with an encircling buckle at the second operation. Only one eye per patient was included, avoiding inter-eye dependence. Anatomical failure was defined conservatively as any subsequent retinal re-detachment, with a separate sensitivity definition for the limited laser-treated event. Principal proportions were reported with confidence intervals, missing data were handled using available case analysis without imputation, and the final analyses were reproduced in R using a fixed validation script. Case-level review also permitted differentiation between re-detachment beneath silicone oil, re-detachment following oil removal, and limited re-detachment managed without further PPV.

The study also has important limitations. First, the retrospective, single-centre design is vulnerable to incomplete documentation, selection bias, and non-standardised follow-up. Twelve eyes lacked documentation of the cause of the first recurrence, and 12 lacked macular status documentation. Follow-up ranged from one to 77 months; consequently, eyes with short follow-up had less opportunity for a subsequent event to be detected, and the crude anatomical success proportion may overestimate longer-term stability. Second, there was no repeat PPV-only comparator, so the study cannot establish whether the encircling buckle improved anatomical or visual outcomes. Third, only six subsequent re-detachment events occurred, limiting the precision of phenotype-specific outcome estimates. Fourth, tamponade and supplementary procedures were selected according to clinical judgement, creating confounding by indication. Fifth, visual acuity measurements were obtained from routine clinical records, and BCVA immediately before the second operation was not consistently available. Finally, PVR grade, axial length, or high-myopia status, detachment extent, and the complete number and location of retinal breaks were not documented consistently enough for adjusted or more granular phenotype analysis.

Within these limitations, the study provides real-world outcome data for a standardised combined approach to first recurrent RRD after primary PPV. Repeat PPV with 360° encircling scleral buckling was followed by anatomical stability without subsequent re-detachment in approximately seven out of eight eyes. The descriptive break location findings suggest that discrete inferior break-related recurrences warrant further comparative study as a potential candidate phenotype for combined surgery. They do not demonstrate that encircling buckling is superior to repeat PPV alone.

Future studies should use multicentre comparative designs with standardised definitions of anatomical success, complete documentation of PVR grade, axial length, detachment extent and retinal break characteristics, and uniform recurrence surveillance. Comparative analyses should specifically evaluate interaction between treatment strategy and recurrence phenotype, particularly inferior break-related disease and PVR severity. Such studies are required to identify which recurrent RRD phenotypes derive incremental benefit from adding an encircling buckle to repeat vitrectomy.

## 5. Conclusions

In this retrospective cohort, repeat PPV combined with 360° encircling scleral buckling was followed by freedom from subsequent retinal re-detachment in 87.2% of eyes. Post hoc descriptive findings were particularly favourable among break-related first recurrences involving inferior, inferonasal, or inferotemporal breaks, but the single-arm design cannot establish that these eyes derive incremental benefit from the buckle. Comparative studies with standardised phenotype-level documentation are needed to guide selection between repeat PPV alone and combined PPV with encircling buckling.

## Figures and Tables

**Table 1:** Baseline and surgical characteristics of the cohort.

Characteristic	Value
Eyes/patients	47/47
Age at Surgery 2, median (IQR); range, years	57.6 (52.6–67.2); 36.7–81.4
**Operated eye**	
Right	26 (55.3%)
Left	21 (44.7%)
Interval from Surgery 1 to Surgery 2, median (IQR); range, days	42 (25.5–63.5); 10–940
**Lens status before Surgery 2**	
Pseudophakic	19/36 (52.8%)
Phakic	17/36 (47.2%)
Missing	11/47 (23.4%)
**Documented cause or contributing factor for RD2**	
Retinal break-related	18/35 (51.4%)
Proliferative vitreoretinopathy	14/35 (40.0%)
Other documented cause	2/35 (5.7%)
Non-adherence to postoperative positioning	1/35 (2.9%)
Missing	12/47 (25.5%)
**Break location among break-related recurrences (n = 18)**	
Inferior/inferonasal/inferotemporal	12/18 (66.7%)
Nasal	2/18 (11.1%)
Superior/superonasal/superotemporal	3/18 (16.7%)
Location not specified	1/18 (5.6%)
**RD2 macular status**	
Macula-on	19/35 (54.3%)
Macula-off	16/35 (45.7%)
Missing	12/47 (25.5%)
**Tamponade at Surgery 1**	
C3F8	32/46 (69.6%)
SF6	11/46 (23.9%)
Silicone oil	3/46 (6.5%)
Missing	1/47 (2.1%)
**Tamponade at Surgery 2**	
C3F8	23/47 (48.9%)
Silicone oil	24/47 (51.1%)
Follow-up after Surgery 2, median (IQR); range, months	13.0 (6.5–24.0); 1–77

Data are presented as n/N (%) unless otherwise stated, where N is the number of eyes with available data. Missing observations are shown separately using the full cohort denominator of 47 eyes. “Retinal break-related” includes documented tears, holes, and inadequately treated retinal breaks. Other documented causes comprised choroidal effusion (n = 1) and macular hole (n = 1), as recorded in the clinical record. Non-adherence to positioning was recorded as a behavioural contributing factor rather than an anatomical cause. Percentages may not total 100% because of rounding.

**Table 2:** Anatomical and visual outcomes after repeat PPV with encircling scleral buckling.

Outcome	Result	95% CI or Range
**Primary anatomical outcome**		
Freedom from any subsequent retinal re-detachment	41/47 (87.2%)	74.8–94.0%
Sensitivity definition excluding the limited laser-treated re-detachment	42/47 (89.4%)	77.4–95.4%
**Re-detachment timing and management**		
Known time to subsequent retinal re-detachment, median (IQR), months	6.0 (2.0–8.0)	2–33; n = 5
Further PPV documented	4/47 (8.5%)	—
Retinectomy documented	4/47 (8.5%)	—
Additional laser retinopexy only	1/47 (2.1%)	—
**Visual outcome**		
Final BCVA, median (IQR), logMAR	0.48 (0.24–0.81)	−0.08 to 2.70
Final BCVA ≤0.30 logMAR	17/47 (36.2%)	—
Final BCVA ≤0.50 logMAR	25/47 (53.2%)	—
Final BCVA ≤1.00 logMAR	41/47 (87.2%)	—
Final BCVA ≤1.30 logMAR	42/47 (89.4%)	—

Subsequent retinal re-detachment was defined as any new area of detached retina after Surgery 2, irrespective of extent or management. The sensitivity definition did not classify the limited re-detachment treated with laser retinopexy alone as surgical failure. Re-detachment timing was unavailable for one of the six events. Final BCVA denotes the last recorded BCVA after Surgery 2.

**Table 3:** Silicone oil outcomes and postoperative complications.

Outcome	Result	Additional Detail
**Silicone oil outcomes**		
Silicone oil used at Surgery 2	24/47 (51.1%)	—
Straightforward silicone oil removal documented	15/24 (62.5%)	Median 4 months (IQR 4–5; range 1–22)
Silicone oil retained at final recorded follow-up	8/24 (33.3%)	—
Complex oil exchange followed by subsequent removal	1/24 (4.2%)	Exchange to heavy silicone oil after subsequent re-detachment
Subsequent retinal re-detachment while silicone oil remained in situ	3/24 (12.5%)	—
Subsequent retinal re-detachment after straightforward silicone oil removal	1/15 (6.7%)	Follow-up after removal was not standardised
**Postoperative complications**		
Any documented clinically relevant complication	11/47 (23.4%)	Categories were not mutually exclusive
Postoperative IOP elevation requiring treatment	7/47 (14.9%)	—
Buckle exposure	4/47 (8.5%)	—
Buckle intervention or removal	5/47 (10.6%)	—
Silicone oil in the anterior chamber	1/47 (2.1%)	Removed after 3 days

Denominators for silicone oil outcomes are restricted to the 24 eyes receiving silicone oil at Surgery 2, except where the full cohort denominator is shown. Complication categories were based on documented events and could overlap within the same eye.

**Table 4:** Exploratory final BCVA subgroup analyses.

Comparison	Group 1, Median (IQR)	Group 2, Median (IQR)	Mann–Whitney *p*
Silicone oil vs. C3F8	0.64 (0.38–0.86)	0.30 (0.14–0.70)	0.018
RD2 macula-off vs. macula-on	0.72 (0.47–0.95)	0.30 (0.18–0.63)	0.011
PVR vs. other documented anatomical cause	0.62 (0.34–1.02)	0.52 (0.24–0.71)	0.214

All final BCVA subgroup analyses were exploratory, unadjusted, and conducted using available cases; no correction for multiple comparisons was applied. For the PVR analysis, eyes with a missing anatomical cause and the eye with non-adherence to positioning as the only documented contributing factor were excluded. For the macular status analysis, 12 eyes with missing RD2 macular status were excluded. The silicone oil comparison is particularly susceptible to confounding by indication.

## Data Availability

The data are not publicly available because they derive from confidential clinical records. De-identified data may be available from the corresponding author upon reasonable request, subject to institutional information-governance approval.

## Supplementary Materials

The following supporting information can be downloaded at: https://www.mdpi.com/article/10.3390/jcm15176801/s1, Table S1: Clinical details of the six subsequent retinal re-detachments.
