# Supplementary material for: Anatomical and Functional Outcomes of Repeat Vitrectomy Combined with Encircling Scleral Buckling for First Recurrent Rhegmatogenous Retinal Detachment After Primary Vitrectomy: A Retrospective Cohort Study
Source: J Clin Med. 2026 Sep 2;15(17):6801. doi: 10.3390/jcm15176801 (PMC13566681; doi:10.3390/jcm15176801)
Supplement: Supplementary file 1 [file jcm-15-06801-s001.zip › jcm-4468386-supplementary.pdf]

Supplementary Table S1. Clinical details of the six subsequent retinal re-detachments.

Event	Documented cause at RD2	Tamponade at Surgery 2	Time to re-detachment, months	Re-detachment context	Subsequent management	Final BCVA, logMAR	Follow-up, months
1	Retinal break-related	C3F8	2	Limited re-detachment	Additional laser retinopexy only	0.00	42
2	PVR	Oil	8	Inferior re-detachment under silicone oil	Further PPV, retinectomy and exchange to heavy silicone oil	0.48	17
3	PVR	Oil	6	PVR re-detachment after ROSO	Further PPV, retinectomy and silicone oil	0.82	31
4	Missing	C3F8	33	Cause not documented	Further PPV, retinectomy and gas	0.12	67
5	PVR	Oil	2	PVR re-detachment under silicone oil	Further PPV, retinectomy and silicone oil	0.68	23
6	PVR	Oil	Missing	Inferior re-detachment under silicone oil	Not documented	1.08	11

Event numbers are study-specific and do not contain patient identifiers. The time to subsequent retinal re-detachment was derived from the available clinical documentation and was missing for one eye. Final BCVA was measured at the last recorded follow-up and may reflect the effect of additional retinal procedures.
